# Are Dutch adults equally susceptible to nudging and pricing strategies? Secondary analyses of the Supreme Nudge parallel cluster-randomised controlled supermarket trial

**DOI:** 10.1186/s12916-024-03429-5

**Published:** 2024-06-10

**Authors:** Josine M. Stuber, Joline W. J. Beulens, Juul J. E. van Lierop, Esmee Schuurman, Jeroen Lakerveld, Joreintje D. Mackenbach, the SUPREME NUDGE consortium, Jody C. Hoenink, Femke Rutters, Wilma E. Waterlander, Denise T. D. de Ridder, Marleen Gillebaart, Stephanie Blom, Femke E. de Boer, Gert-Jan de Bruijn, Anne L. Vos, Edith G. Smit, Michel C. A. Klein, Jacqueline E. W. Broerse, Tjerk-Jan Schuitmaker-Warnaar, Cédric N. H. Middel, Yvonne T. van der Schouw, Ivonne Sluijs, Marjolein C. Harbers, Elizabeth Velema

**Affiliations:** 1 Epidemiology and Data Science, Amsterdam UMC Location Vrije Universiteit Amsterdam, De Boelelaan 1117, Amsterdam, The Netherlands; 2 Amsterdam Public Health, Amsterdam, The Netherlands; 3 Upstream Team, www.upstreamteam.nl, Amsterdam UMC Location Vrije Universiteit Amsterdam, De Boelelaan 1117, Amsterdam, Netherlands; 4 Julius Center for Health Sciences and Primary Care, University Medical Center Utrecht, Utrecht University, Universiteitsweg 100, Utrecht, The Netherlands

**Keywords:** Prevention, Food environment, Choice architecture, Public Health Nutrition, Grocery store

## Abstract

**Background:**

Supermarket interventions are promising to promote healthier dietary patterns, but not all individuals may be equally susceptible. We explored whether the effectiveness of nudging and pricing strategies on diet quality differs by psychological and grocery shopping characteristics.

**Methods:**

We used data of the 12-month Supreme Nudge parallel cluster-randomised controlled supermarket trial, testing nudging and pricing strategies to promote healthier diets. Participants were Dutch speaking adults aged 30–80 years and regular shoppers of participating supermarkets (*n* = 12) in socially disadvantaged neighbourhoods. Data on psychological characteristics (food-related behaviours; price sensitivity; food decision styles; social cognitive factors; self-control) and grocery shopping characteristics (time spent in the supermarket; moment of the day; average supermarket visits; shopping at other retailers; supermarket proximity) were self-reported at baseline. These characteristics were tested for their moderating effects of the intervention on diet quality (scored 0–150) in linear mixed models.

**Results:**

We included 162 participants from intervention supermarkets and 199 from control supermarkets (73% female, 58 (± 10.8) years old, 42% highly educated). The interventions had no overall effect on diet quality. Only five out of 23 potential moderators were statistically significant. Yet, stratified analyses of these significant moderators showed no significant effects on diet quality for one of the subgroups and statistically non-significant negative effects for the other. Negative effects were suggested for individuals with lower baseline levels of meal planning (*β* − 2.6, 95% CI − 5.9; 0.8), healthy shopping convenience (*β* − 3.0, 95% CI − 7.2; 1.3), and healthy food attractiveness (*β* − 3.5, 95% CI − 8.3; 1.3), and with higher levels of price consciousness (*β* − 2.6, 95% CI − 6.2; 1.0) and weekly supermarket visits (*β* − 2.4, 95% CI − 6.8; 1.9).

**Conclusions:**

Adults with varying psychological and grocery shopping characteristics largely seem equally (un)susceptible to nudging and pricing strategies. It might be that certain characteristics lead to adverse effects, but this is not plausible, and the observed negative effects were small and statistically non-significant and may be explained by chance findings. Verification of these findings is needed in real-world trials based on larger sample sizes and with the use of more comprehensive interventions.

**Trial registration:**

Dutch Trial Register ID NL7064, 30th of May, 2018, https://onderzoekmetmensen.nl/en/trial/20990

**Supplementary Information:**

The online version contains supplementary material available at 10.1186/s12916-024-03429-5.

## Background

Chronic diseases, such as cardiovascular diseases and type 2 diabetes, are highly prevalent and the leading causes of death globally [1, 2]. It is well established that adhering to a healthy dietary pattern can reduce chronic disease risk [3–7], and supermarket nudging and pricing strategies have frequently been proposed as solutions to promote healthier population diets. Nudging refers to environmental changes that promote a certain choice without removing the alternative choice [8]. Examples of supermarket nudges are replacements of confectionery products at the check-out counter by healthy snacks, or placing healthier products on eye-level. Evidence from systematic reviews in real-world purchasing settings demonstrates that nudges have, on average, modest but positive effects on promoting healthy product purchases [9–11]. Pricing strategies to promote healthy products are likely most effective when implemented as price decreases on healthy products and price increases on unhealthy products [12, 13]. The most well-known pricing strategy is a sugar tax to discourage sugar-sweetened beverage purchases, which has proven to be effective [14, 15].

Nudging and pricing strategies target different decision-making processes of supermarket customers. Based on the dual-process theory, decisions can be divided into automatic and fast decisions (referred to as System 1), and controlled and slow decisions (System 2) [16]. Nudges target System 1, for example, by making certain products more noticeable to trigger subconscious grocery decisions. Pricing strategies likely tap into System 2, as they require individuals to deliberately (re)consider the price in their purchasing decision. When pricing strategies are combined with promotional signs it can be seen as a nudge on top of the pricing strategy, thus relying on both System 1 and System 2. In that case, System 1 (promotional sign attracts attention) may trigger activation of System 2 ((re)considering the price), and their combination may yield the largest effects on purchasing decisions.

It is likely that not all individuals are equally susceptible to nudging and pricing strategies, as certain psychological characteristics may interact with potential nudging and pricing effects. For example, higher levels of price sensitivity might lead to increased activation of System 2 and subsequent higher susceptibility to pricing strategies, while lower levels of price sensitivity might result in higher reliance on System 1 and thus higher nudge susceptibility. Theory indicates that those with a low motivation for a certain message, low habit strength, or higher levels of impulsivity may rely more on System 1 leading to higher nudge susceptibility [17, 18]. On the other hand, high levels of health consciousness, habit strength, and strong product preferences may inhibit the activation of System 1, and thus attenuate nudge susceptibility [19]. Other characteristics such as higher levels of cooking skills or meal planning might also attenuate nudge susceptibility, since having the higher levels of these characteristics likely enhances relying on System 2.

The limited research on this topic thus far shows higher nudge susceptibility in those with low habit strength [20], low self-control [21], and non-dieters [22], and among those giving low priority to weight control [23]. Moreover, research suggests that price changes are equally effective across different levels of impulsivity, financial constraint, perceived stress, price sensitivity, intuitive and spontaneous decision-making styles, but more effective for those who give low priority to the food choice motives “natural content of foods” and “weight control” [23–25]. In addition, only one study thus far explored the moderating effects of various psychological characteristics for the combination of nudging and pricing strategies. It suggested equal positive effects on healthier purchasing outcomes across individuals with differing levels of impulsivity, price sensitivity, intuitive and spontaneous decision-making styles, and food choice motives [23]. However, this study was conducted in an experimental virtual supermarket environment and it remains to be determined whether these observations can be translated to real-world purchasing settings.

Whether variation in grocery shopping habits of customers may moderate nudging and pricing effectiveness is currently unclear. For example, some grocery shopping characteristics such as spending more time in the supermarket, shopping multiple times per week, and less frequent shopping at other food retailers may lead to higher exposure to interventional strategies, which potentially increases their effectiveness.

Equity-promoting effects, potentially with the largest effects in the most vulnerable groups, of nudging and pricing strategies across individuals with different characteristics would be desirable. It would indicate that the investigated subgroups could all benefit while reducing inequity, or are all equally unaffected, and provide insight into whether nudging and pricing strategies are suitable as general health-promoting strategies. Also, it is important to know if nudging and pricing strategies potentially lead to adverse effects (i.e. lower diet quality) among certain subgroups, which could hinder recommendations to implement these strategies on a large scale.

In our earlier work, we have shown that co-created supermarket nudging and pricing strategies were unable to change diet quality and that intervention effects were modified by age but not educational attainment and sex [26]. We now explore whether and how the effectiveness of nudging and pricing strategies on diet quality may differ by various psychological and grocery shopping characteristics, among Dutch adults in a real-world supermarket setting.

## Methods

### Trial design

For these secondary analyses, we used data from the Supreme Nudge parallel cluster-randomised controlled supermarket trial [27, 28]. The Supreme Nudge trial was implemented in 12 supermarkets located in socially disadvantaged neighbourhoods across the Netherlands. Stores were randomised to either the control group (*n* = 6) or the intervention group receiving nudging and pricing strategies to promote healthy products (*n* = 6). The trial ran from 6 months (*n* = 4) to 1 year (*n* = 8), depending on supermarket enrolment date.

### Participants

Participant inclusion criteria were as follows: aged 30–80 years at the time of registration, living in the socially disadvantaged neighbourhood of a participating supermarket, being a regular shopper at a participating supermarket (> 50% weekly groceries at a selected store), planning on continuing shopping there for the study period, and having the ability to communicate in Dutch.

### Recruitment

A stepwise participant recruitment strategy was used [29]. Recruitment started with passive strategies, including online news articles in local newspapers, supermarket flyers, e-mail invites to supermarket customer panels, in-store posters and other locations surrounding the supermarket (e.g. physiotherapist practices), postal invitation letters to every household in the supermarkets’ neighbourhoods, advertisement on the website of the study funder (Dutch Heart Foundation), and a social media campaign. Next, active recruitment strategies included asking participants to invite their partner or neighbours for participation, and in-store recruitment by the research team.

### Interventions

Detailed information on the applied interventions is previously published [26]. Briefly, the supermarket interventions consisted of nudging and pricing strategies to promote healthier purchasing, which were developed according to a co-creative process with supermarket stakeholders and interventionists [27, 30]. Nudging and pricing strategies promoted healthy food groups which are recommended in the Dutch dietary guidelines [31]. Applied nudges on healthy products targeted 9% of the supermarket assortment and can be divided into placement nudges, focusing on availability and position, and into property nudges, focusing on presentation and information [32]. Placement nudges consisted of increased shelf space of healthier products and changing their location in the store. The property nudges focused on information symbols which highlighted the product’s *tastiness*, *convenience* or *popularity*, which were also used on different forms of promotional materials. Pricing strategies targeted 3% of the supermarket assortment and consisted of price decreases of healthy products and price increases of unhealthy products. Wherever possible, pricing strategies were implemented within the same food group. Price decreases were − 25%, or − 10% when combined with price increases (+ 15%) in the same food group. For example, fresh fruit and vegetables were − 25%, and whole-grain pasta products were − 10% with a simultaneous + 15% on the non-whole grain pasta (Additional file 1: Table S1).

### Randomisation and blinding

The 12 supermarkets were cluster-randomised by the research team to the control group or the intervention group via a web-based random number generator tool in blocks of four. Blinding of participants was not possible due to the nature of the nudging and pricing strategies, Yet, participants were not, prior to participation nor during the study, actively informed of their supermarket allocation.

### Data collection

Measurements took place at baseline (T0), after 3 months (T1), 6 months (T2), and 12 months (T3). Questionnaires were used to collect data on diet quality (T0, T1, T2, and T3) [33, 34], population characteristics (T0), and psychological characteristics and grocery shopping characteristics (T0) as potential moderators: food-related behaviours [35], price sensitivity [36], food decision styles [37–41], social cognitive factors related to healthy eating [42–47], self-control [48], time spent in the supermarket, moment of the day grocery shopping, average weekly supermarket visits, shopping at other food retailers, and supermarket proximity. Details on data collection methods and validity of used measurements have been previously described [27, 28], and a summary is provided in Table 1.

**Table 1:** Collected data and their operationalisation

Collected data	Operationalisation
***Population characteristics***	
Age	Age in years at time of study registration
Sex	Male; female
Educational attainment	Low educational attainment (no education, primary school); medium educational attainment (secondary educational attainments); high educational attainment (tertiary/higher educational attainments)
***Outcome***	
Diet quality	A short 40-item Dutch Healthy Diet food frequency questionnaire was used to measure diet quality (“Dutch Healthy Diet 2015 index score”) of 15 food group components, which were each scored from 0 to 10, resulting in a total diet quality score ranging from 0 (low diet quality) to 150 (high diet quality)
***Psychological characteristics***	
Food-related behaviours	Four constructs which each used 9 items: conscious grocery shopping (e.g. use of shopping list), conscious eating (e.g. have dinner at the table with family), meal planning (e.g. preparing meals in advance), and cooking skills (e.g. use of new ingredients). Items were scored from 0–3 (never, sometimes, usually, always) as well as a response option ‘not applicable’. For each construct, the mean score of all items was calculated. The response option ‘not applicable’ was treated as a missing value. When participants had ≥ 4 out of 9 items missing on one construct, the calculated mean score was recoded into missing
Price sensitivity	Three constructs: price consciousness (5 items, e.g. paying more attention to prices), sale proneness (6 items, e.g. proneness to buy price-promoted products), and value consciousness (7 items, e.g. aiming to maximize product quality for a certain price). Responses were rated on 7-point Likert scales ranging from 1 to 7 (strongly disagree – strongly agree). Mean scores were calculated for each construct
Food decision styles	Three food decision style constructs (for vegetables and for snacks): reflective (e.g. choosing products attentively), habitual (e.g. choosing on autopilot mode), and impulsive (e.g. choosing spontaneously), each containing 5 items measured on a 7-point Likert scale (strongly disagree–strongly agree). Mean scores were calculated for each construct Items on food decision styles were preceded by asking if the participant was used to purchase fruits and vegetables/snacks in the supermarket on a regular basis (yes, no). If participants indicated ‘no’, the items on food decision styles related to fruits and vegetables and/or snack were not shown and thus treated as missing values
Social cognitive factors related to healthy eating	Four constructs, including health goals (1 item: ‘I think it’s important to eat healthy’), experienced convenience of healthy grocery shopping (2 items, e.g. ‘healthy products are available in my supermarket’), perceived social norm (2 items, e.g.’my friends and family eat healthy’), and healthy food attractiveness (1 item:’healthy products are tasty’). Items were rated on 7-point Likert scales (strongly disagree–strongly agree) and mean scores were calculated for the constructs convenience of healthy grocery shopping and perceived social norm
Self-control	One item via a revised version of the self-control ladder measuring self-perceived discipline levels on a 10-point Likert scale (no perceived discipline–high levels of perceived discipline)
***Grocery shopping characteristics***	
Time spent in the supermarket	Four categories (less than 15 min, 15 min to half an hour, half an hour to an hour, longer than an hour) were recoded into less than 15 min and in more than 15 min, based on the number of participants in each response category to create a balanced dichotomous variable for interaction testing
Moment of the day grocery shopping	Three categories (morning, midday, evening) which were dichotomised into to mainly in the morning, and mainly in the midday or evening, based on the number of participants in each response category to create a balanced dichotomous variable
Average weekly supermarket visits	The number of days per week a participant visited a supermarket on average
Shopping at other food retailers	The total number of times in the past two weeks a participant visited for example a bakery, farmer markets, or butcher shop
Supermarket proximity	Meters distance from the participants’ home address to their participating study supermarket

### Statistical analyses

Descriptive statistics for the sociodemographic variables, diet quality score at T0 and the potential moderators were reported separately by the trial arm. Continuous variables with normal distributions were reported by their mean and standard deviation (SD), or by the median and interquartile range (IQR) in case of non-normality. Dichotomous and categorical variables were described by frequencies and percentages.

We used linear mixed models with group allocation as the independent variable and the diet quality score at T1, T2 and T3 as the dependent variable, including diet quality at T0 and time (categorical) as covariates. All models included random intercepts on the participant and on the supermarket level, based on intra-cluster correlation coefficients (ICCs) > 0 in the crude model without an interaction term. Interactions were tested between the group allocation and all potential moderators (food-related behaviours, price sensitivity, food decision styles, social cognitive factors related to healthy eating, self-control, and grocery shopping characteristics).

We used all available data of those participants who completed the baseline questionnaire, and the baseline measurement of diet quality with at least one follow-up measurement. Participants with missing data on certain moderators were excluded from the analysis involving those specific moderators. The absence of 0 in the 90% confidence interval (CI) was deemed a significant interaction considering the original sample size of the Supreme Nudge trial was not powered for subgroup analyses [27]. We did not account for multiple testing since our analyses were pre-planned in our protocol paper [27] and are of an explorative nature [49].

We report the regression coefficients (*β*) and 90% CI’s of the interaction terms as study outcomes, and stratified results are presented for significant moderators. Stratified subgroups were created according to the median, in which the median value was used in the upper category of each subgroup comparison. Effects within subgroups are reported by regression coefficients and 95% CI’s, and not 90% CI’s, to provide insight into the robustness of the findings.

Analyses were performed in R statistical software (version 4.3.2) using the *lme4* package.

## Results

In total, 361 participants completed the baseline questionnaire and at least one of the follow-up measurements of diet quality (Additional file 1: Figure S1). The sociodemographic characteristics of participants were equally distributed between the control group (*n* = 199) and the intervention group (*n* = 162). The study sample consisted of 73% females, with a mean age of 58 (± 10.8) years, and 42% were highly educated. Mean scores on diet quality and all potential moderators were comparable between the groups (Table 2). In both groups, an approximately even proportion of participants shared a household, with 12% (*n* = 24) in the control group and 9% (*n* = 14) in the intervention group. The ICCs were 0.39 and 0.04 for the clustering of data within participants and of participants within supermarkets, respectively.

**Table 2:** Study population baseline characteristics of the Supreme Nudge trial (*n* = 361)

	Control group (*n* = 199)		Intervention group (*n* = 162)	
Sex, females	142	(71.4)	120	(74.1)
Age in years^a^	57.2	± 10.2	58.9	± 11.5
Educational attainment				
*Low*	38	(19.1)	46	(28.4)
*Medium*	76	(38.2)	50	(30.9)
*High*	85	(42.7)	66	(40.7)
Diet quality (Dutch Healthy Diet 2015 index) at T0, scored from 0 (low) to 150 (high)	106.0	± 18.3	103.9	± 19.4
Food-related behaviours, scored from 0 (never) to 3 (always)				
*Conscious grocery shopping*^b^	1.5	± 0.3	1.5	± 0.4
*Conscious eating*^c^	2.3	± 0.4	2.2	± 0.5
*Cooking skills*^d^	1.7	± 0.4	1.6	± 0.4
*Meal planning*^b^	2.5	± 0.3	2.5	± 0.3
Price sensitivity^b^, scored from 1 (low) to 7 (high)				
*Price consciousness*	3.9	± 1.5	3.8	± 1.5
*Sale proneness*	4.7	± 1.2	4.7	± 1.2
*Value consciousness*	5.0	± 1.1	5.0	± 1.1
Food decision styles for fruit and vegetables^e^, scored from 1 (low) to 7 (high)				
*Reflective*	5.3	± 1.2	5.2	± 1.1
*Habitual*	5.0	± 0.9	4.9	± 0.9
*Impulsive*	3.4	± 1.2	3.5	± 1.2
Food decision styles for snacks^f^, scored from 1 (low) to 7 (high)				
*Reflective*	4.0	± 1.4	3.8	± 1.3
*Habitual*	3.1	± 1.3	3.2	± 1.4
*Impulsive*	3.6	± 1.6	3.7	± 1.4
Social cognitive factors related to healthy eating^b^, scored from 1 (low) to 7 (high)				
*Health goals*	6.4	± 0.9	6.3	± 0.8
*Healthy shopping convenience*	5.9	± 1.0	6.1	± 0.9
*Perceived social norm*	4.7	± 0.9	4.7	± 1.0
*Healthy food attractiveness*	5.9	± 1.1	5.9	± 1.2
Self-control, scored from 1 (low) to 10 (high)	6.9	± 1.5	6.9	± 1.5
Grocery shopping characteristics^a^				
*Time spent in the supermarket*				
Less than 15 min	35	(17.6)	36	(22.2)
More than 15 min	164	(82.4)	125	(77.2)
*Moment of the day grocery shopping*				
Mostly in the morning	98	(49.2)	72	(44.4)
Mostly in the afternoon or evening	101	(50.8)	89	(54.9)
*Average number of weekly supermarket visits*	3.2	± 1.5	3.3	± 1.5
*Shopping at other food retailers in the past 2 weeks (number of times)*	3.0	[2.0]	3.0	[3.0]
*Supermarket proximity in meters*	800.0	[950.0]	675.0	[863.0]

Values represent: *n* (percentage); mean ± standard deviation; median [interquartile range]; low educational attainment = no education and primary education, medium educational attainment = secondary educational attainments, high educational attainment = tertiary educational attainments

^a^*n* = 1 missing; ^b^*n* = 2 missing; ^c^*n* = 12 missing; ^d^*n* = 7 missing; ^e^*n* = 27 missing; ^f^*n* = 127 missing

As previously reported, the nudging and pricing strategies had no overall effect on diet quality (*β* − 1.1 (95% CI 3.8 to 1.7)) [26]. Most of the explored moderators did not show significant interaction effects with the intervention group, but five out of 23 did (Table 3). Compared to control participants, participants in the intervention group had for each unit increase in the baseline levels of meal planning a 6-point (90% CI 0.2 to 11.5) higher diet quality after 12 months of follow-up. Moreover, a higher diet quality was observed for participants with each unit increase in experienced healthy shopping convenience (*β* 1.9 (0.2 to 3.6)), and healthy food attractiveness (*β* 1.9 (0.4 to 3.3)), and for each unit decrease in price consciousness (*β* − 1.2 (− 2.2 to − 0.1)), and average number of weekly supermarket visits (*β* − 1.6 (− 2.7 to − 0.5)).

**Table 3:** Interaction between nudging and pricing strategies and potential moderators on diet quality (*n* = 361)

	***β***	90% CI
Food-related behaviours		
*Nudging and pricing strategies* × *Conscious grocery shopping*^*a*^	0.1	− 5.2 to 4.9
*Nudging and pricing strategies* × *Conscious eating*^*b*^	− 0.6	− 4.5 to 3.2
*Nudging and pricing strategies* × *Cooking skills*^*c*^	0.4	− 4.4 to 4.7
*Nudging and pricing strategies* × *Meal planning*^*a*^	**6.0**	**0.2 to 11.5**
Price sensitivity^a^		
*Nudging and pricing strategies* × *Price consciousness*	** − 1.2**	** − 2.2 to − 0.1**
*Nudging and pricing strategies* × *Sale proneness*	0.2	− 1.2 to 1.6
*Nudging and pricing strategies* × *Value consciousness*	− 0.0	− 1.4 to 1.5
Food decision styles for fruit and vegetables^d^		
*Nudging and pricing strategies* × *Reflective*	− 0.1	− 1.7 to 1.4
*Nudging and pricing strategies* × *Habitual*	1.4	− 0.5 to 3.3
*Nudging and pricing strategies* × *Impulsive*	0.7	− 0.7 to 2.2
Food decision styles for snacks^e^		
*Nudging and pricing strategies* × *Reflective*	− 0.0	− 1.5 to 1.4
*Nudging and pricing strategies* × *Habitual*	1.1	− 0.3 to 2.5
*Nudging and pricing strategies* × *Impulsive*	− 0.9	− 2.1 to 0.4
Social cognitive factors related to healthy eating^a^		
*Nudging and pricing strategies* × *Health goals*	0.8	− 1.2 to 2.8
*Nudging and pricing strategies* × *Healthy shopping convenience*	**1.9**	**0.2 to 3.6**
*Nudging and pricing strategies* × *Perceived social norm*	0.9	− 0.8 to 2.6
*Nudging and pricing strategies* × *Healthy food attractiveness*	**1.9**	**0.4 to 3.3**
Self-control		
*Nudging and pricing strategies* × *Self-control*	0.4	− 0.7 to 1.5
Grocery shopping characteristics^f^		
*Nudging and pricing strategies* × *More than 15 min spent in the supermarket*	1.0	− 3.0 to 5.3
*Nudging and pricing strategies* × *Grocery shopping mostly in the afternoon or evening*	− 1.1	− 4.4 to 2.2
*Nudging and pricing strategies* × *Average number of weekly supermarket visits*	** − 1.6**	** − 2.7 to − 0.5**
*Nudging and pricing strategies* × *Shopping at other food retailers in past two weeks*	0.5	− 0.4 to 1.3
*Nudging and pricing strategies* × *Supermarket proximity*	0.0	− 0.0 to 0.0

Analyses were based on linear mixed models with random intercepts for supermarket locations and for participants, including diet quality at baseline and time as covariates, and the control group was used as reference category in all analyses

Bold values indicate statistical significance (*p* < 0.10)

^a^*n* = 2 missing; ^b^*n* = 12 missing; ^c^*n* = 7 missing; ^d^*n* = 27 missing; ^e^*n* = 127 missing; ^f^*n* = 1 missing

Stratified analyses of significant moderators showed, for all moderators, no effects for one of the subgroups and a modest and statistically non-significant negative effect on diet quality for the other (Table 4). Negative effects were suggested for individuals with lower baseline levels of meal planning, experienced healthy shopping convenience, and healthy food attractiveness, and with higher levels of price consciousness and weekly supermarket visits. For example, for participants characterised as having lower levels of meal planning, intervention exposure led to a − 2.6 points (95% CI − 5.9 to 0.8) lower diet quality compared to the control group, while for those characterised as having higher levels of meal planning intervention exposure did not change diet quality (*β* − 0.1 (− 2.8 to 2.9)) compared to the control group.

**Table 4:** Stratified analyses of significant moderators of nudging and pricing strategies on diet quality (*n* = 361)

	*β*	95% CI
Meal planning^a^		
*Lower level of meal planning (scores 0.0–2.4), n* = *173*	− 2.6	− 5.9 to 0.8
*Higher levels of meal planning (scores 2.5–3.0), n* = *186*	− 0.1	− 2.8 to 2.9
Price consciousness^a^		
*Lower levels of price consciousness (scores 1.0–3.7), n* = *171*	0.3	− 2.6 to 3.2
*Higher levels of price consciousness (scores 3.8–7.0) n* = *188*	− 2.6	− 6.2 to 1.0
Healthy shopping convenience^a^		
*Lower levels of experienced healthy shopping convenience (scores 1.0–5.9), n* = *112*	− 3.0	− 7.2 to 1.3
*Higher levels of experienced healthy shopping convenience (scores 6.0–7.0), n* = *247*	− 0.3	− 3.4 to 2.8
Healthy food attractiveness^a^		
*Lower levels of healthy food attractiveness (scores 1.0–5.9), n* = *102*	− 3.5	− 8.3 to 1.3
*Higher levels of healthy food attractiveness (scores 6.0–7.0), n* = *257*	− 0.4	− 2.9 to 2.3
Average number of weekly supermarket visits^b^		
*Lower number of weekly supermarket visits (scores 0–2), n* = *127*	0.4	− 2.9 to 3.7
*Higher number of weekly supermarket visits (scores 3–7)), n* = *233*	− 2.4	− 6.8 to 1.9

Analyses were based on linear mixed models with random intercepts for supermarket locations and for participants, including diet quality at baseline and time as covariates, and the control group was used as reference category in all analyses

^a^*n* = 2 missing; ^b^*n* = 1 missing

## Discussion

This study showed that the effect of nudging and pricing strategies in real-world supermarkets on diet quality was not moderated by most of the explored psychological and grocery shopping characteristics. In addition, findings suggest a modest negative effect on diet quality after exposure to nudging and pricing strategies for individuals with lower baseline levels of meal planning, healthy shopping convenience, and experienced healthy food attractiveness, and with higher baseline levels of price consciousness and number of weekly supermarket visits.

Most studies on the moderating effects of psychological characteristics investigated the isolated effect of nudging or pricing strategies [18, 20–22, 24, 50]. We used a combined intervention of nudging and pricing strategies for which the theoretical foundation differs: nudging relies on unconscious decisions while pricing strategies rely more on conscious decisions [16]. Nonetheless, our results build upon the earlier findings from a simulated virtual supermarket experiment in which the investigated psychological characteristics impulsivity, price sensitivity, decision-making styles, and food choice motives did not moderate the effect of nudging and pricing strategies [23]. Our findings are also in line with the previous observation that healthy eating motivation does not seem to be a moderator [50]. However, in contrast to previous observations from simulated study settings [17, 18, 20, 21, 24], we did not observe that lower levels of self-control, conscious grocery shopping, and habit strength led to higher intervention susceptibility in a real-world setting. The influence of variation in psychological characteristics across individuals and their response to the nudging and pricing strategies may be overruled by the many other (marketing) stimuli present in a real-world supermarket setting which are driving purchasing behaviours [51]. It may also be overruled by strong product preferences or habitual shopping patterns in the supermarket which individuals may use to minimise the time and mental capacity required for grocery shopping. In the present study, we had limited insight in habitual shopping patterns. Indeed, we measured habitual shopping related to vegetables and snack purchases, but, for example, not to other food groups or specific brands, or overall grocery shopping habits (e.g. use of grocery lists or habitual store routing).

The potentially negative effects of nudging and pricing strategies on diet quality among the five statistically significant moderators are not easily explained. It might be that certain characteristics lead to adverse effects, but this is not plausible and it should be noted that the observed negative effects were small and statistically non-significant, and may be explained by chance findings. Verification of these findings is thus crucial. Nevertheless, it might be that those with lower levels of experienced convenience of healthy shopping and experienced healthy food attractiveness somewhat oppose of healthier products or do not want to be patronised by the nudging and pricing strategies. Lower diet quality for those with higher levels of price consciousness may be explained by having a lower income to spend on groceries compared to those with lower price consciousness. Lower diet quality among those with more frequent supermarket visits per week in general — not specifically to a participating study supermarket — might be due to lower intervention exposure in number of minutes per visit and throughout the different supermarket sections, as more frequent visits may mean more visits to different supermarket chains.

The observations that most of the investigated moderators did not influence susceptibility to nudging and pricing strategies and that significant moderators showed relatively small effect sizes are promising for these interventions as a general public health strategy. It will likely not increase dietary inequalities between subgroups with varying psychological and grocery shopping characteristics. Verification of our findings is needed in future real-world randomised controlled supermarket trials based on larger sample sizes and with the use of more comprehensive intervention strategies. Strategies should, besides promoting healthy purchases, discourage unhealthy purchases (e.g. no marketing and promotions of unhealthy products, elimination of unhealthy products at checkouts, and limited availability of unhealthy products) [52, 53]. Moreover, future real-world trials should further investigate the equity of nudging and pricing strategies by investigating sociodemographic characteristics such as income and ethnicity as moderators. Especially income is a known moderator for pricing strategies, but whether low or high-income groups are more responsive to pricing strategies is not consistently shown [24]. Lastly, to create impactful and sustainable changes in supermarkets, there is an urgent need for policy measures that create a level playing field among food retailers to overcome commercial barriers that currently hinder impactful changes [54].

### Strengths and limitations

This study was based on a strong cluster-randomised and controlled trial design, securing a high internal validity. It was the first of its kind by investigating the combination of nudging and pricing strategies and potential moderating factors in a real-world purchasing setting. In addition, the longitudinal design enabled us to determine the moderation effects based on within-subject and between-subject average changes in diet quality. However, some limitations should be acknowledged. First, our study power was likely relatively low as the original sample size of the Supreme Nudge trial was not powered for subgroup analyses, although exploring for effect modification was planned in our study protocol [27]. We aimed to address the lack of power via the use of a 90% CI to determine significant interactions, which could have led to a less precise estimation of interaction effects. Second, the external validity may be limited due to a 10 points higher diet quality score at baseline than is observed in a cross-sectional Dutch population sample [55]. This may have attenuated the intervention effects and thus the ability to detect potential moderators. Third, measurement of dietary intake via quantitative questionnaires is prone to over- or underreporting and socially desirable answers. The diet quality score based on the short 40-item food frequency questionnaire (FFQ) was validated against a 180-item FFQ combined with a 24-h urinary sodium excretion value, which revealed a moderate correlation of 0.56 [33]. Yet, our analyses are likely minimally affected by this measurement error since we adjusted for the diet quality baseline value. Fourth, this intervention combined nudging and pricing strategies but the implementation of nudging versus pricing strategies was outbalanced. As this was a real-world trial, in which the intervention component development was based on a co-creative process with the participating supermarket chain, the number of nudging types and their implementation across various food groups were outweighing the smaller absolute number of implemented pricing strategies — which were based on a maximum of 200 price changes per week. An unequally balanced intervention dosage may have influenced our findings, since nudging and pricing strategies rely on different theoretical foundations and food prices are known to be a strong driver of food purchasing decisions [12].

## Conclusions

Dutch adults with varying psychological and grocery shopping characteristics seem to a large extent equally (un)susceptible to nudging and pricing strategies. Stratified analyses suggested a modest and statistically non-significant negative effect on diet quality after exposure to nudging and pricing strategies for individuals with lower baseline levels of meal planning, healthy shopping convenience, and experienced healthy food attractiveness, and with higher baseline levels of price consciousness and number of weekly supermarket visits. These potential adverse effects are not plausible and the observed negative effects were small and statistically non-significant and may be explained by chance findings. Verification of these findings is needed in future real-world randomised controlled supermarket trials based on larger sample sizes and with the use of more comprehensive intervention strategies.

### Supplementary Information

Additional file 1: Table S1. Overview of implemented supermarket interventions in the Supreme Nudge trial. Figure S1. Participant flowchart.

## Data Availability

The data analysed during the current study are not publicly available as it will violate participant consent. The analysis plan and analytical code are available from the corresponding author on reasonable request.

## Abbreviations

CI: Confidence interval
FFQ: Food frequency questionnaire
