# Supplementary material for: Are Dutch adults equally susceptible to nudging and pricing strategies? Secondary analyses of the Supreme Nudge parallel cluster-randomised controlled supermarket trial
Source: BMC Med. 2024 Jun 10;22:228. doi: 10.1186/s12916-024-03429-5 (PMC11163734; doi:10.1186/s12916-024-03429-5)
Supplement: Supplementary file 1 — Additional file 1: Table S1. Overview of implemented supermarket interventions in the Supreme Nudge trial. Figure S1. Participant flowchart. [file 12916_2024_3429_MOESM1_ESM.docx]

**Are Dutch adults equally susceptible to nudging and pricing strategies? Secondary analyses of the Supreme Nudge parallel cluster-randomised controlled supermarket trial**

*J M Stuber et al*

Additional file 1

[Table S1. Overview of implemented supermarket interventions in the Supreme Nudge trial 2](#_Toc164066875)

[Figure S1. Participant flowchart of the Supreme Nudge trial (n=361) 3](#_Toc164066876)

# Table S1. Overview of implemented supermarket interventions in the Supreme Nudge trial

| **Intervention strategies** | **Components** | **Explanation of intervention** | **Targeted product groups** |
| --- | --- | --- | --- |
| Placement nudges | Healthy shelf layout | Healthy products placed at eye level. | Shelves including pasta and rice products, bread substitutes, and breakfast cereals. |
|  | Healthy checkout | One check-out till presenting only healthy products. | Sugar-free beverages, nuts, snack vegetables, dried fruits, whole-grain crackers, instead of confectionery products. |
|  | Healthy end of aisle | Promoting only healthy products on a prominent end of aisle; monthly product switch. | Whole-grain products, oils, canned fish/legumes/ tomatoes, nuts, natural peanut butter, sugar-free beverages. |
|  | Healthy aisle baskets | Three to four aisle baskets filled with healthy products; monthly product switch. | Nuts, canned fish/legumes/tomatoes, whole-grain products. |
| Property nudges | Shelf-labels | Symbols which highlighted the product’s *tastiness* (smiley symbol), *convenience* (stopwatch symbol indicating ease of preparation) or *popularity* (thumbs-up symbol). | All healthy products across a range of food groups (i.e., vegetables, fruits, all whole-grain products, fish, milk and yogurt, cheese, legumes, butters and oils, nuts, sugar-free bottled beverages, and tea) |
|  | Shelf feedback strips | Positive feedback strip with popularity symbol. | Underneath whole-grain bread, fresh fish and snack vegetables. |
|  | Shelf-stoppers | Shelf cards highlight one healthy product per product group; monthly product switch. | At each shelf including the shelf-labels. |
|  | Healthy suggestions shelf-banners | Seasonal shelf-banners suggesting different healthy product combinations using the convenience symbol. | Whole-grains, legumes and bread substitutes. |
|  | Explanation of nudging symbols | The symbols are introduced on the shopping cart handles, and also shown on shopping cart/baskets boards and the checkout divider bars. | N/A |
| Pricing strategies | Price reductions | Price reductions were -25%, or -10% when combined with price increases in the same food group; three-weekly product switch. | Healthy product groups: vegetables, fruit, whole-grain products, fresh fish, low and medium-fat dairy, low-salt legumes, butters and oils with unsaturated fats, unsalted nuts, sugar-free beverages. |
|  | Price increases | Price increases were +15%; three-weekly product switch. | Unhealthy product groups: non whole-grain products, processed/salted fish, high fat and sugary dairy, high-salt legumes, butters and oils with saturated fats, salted nuts, sugary beverages. |


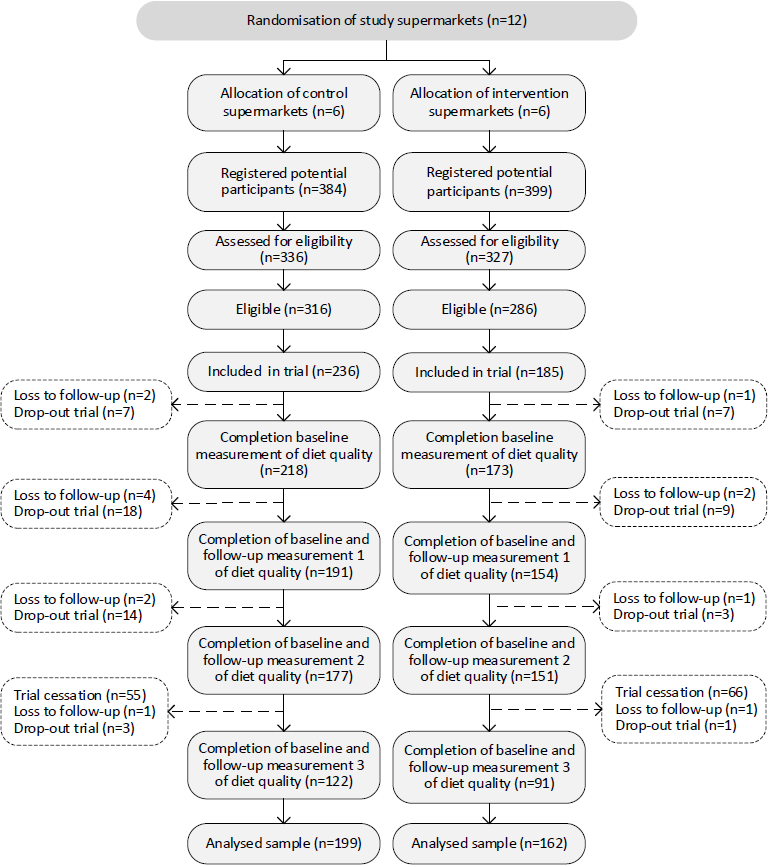


# Figure S1. Participant flowchart of the Supreme Nudge trial (n=361)
